# Multichannel esophageal signals to monitor respiratory rate in preterm infants

**DOI:** 10.1038/s41390-021-01748-4

**Published:** 2021-10-02

**Authors:** Corine Bürgin, Patrizia Simmen, Nishant Gupta, Lilian Suter, Samuel Kreuzer, Andreas Haeberlin, Sven M. Schulzke, Daniel Trachsel, Thomas Niederhauser, Kerstin Jost

**Affiliations:** 1 Department of Pediatrics, University Children’s Hospital Basel UKBB, Basel, Switzerland; 2 Institute for Human Centered Engineering HuCE, Bern University of Applied Sciences, Biel, Switzerland; 3 Department of Cardiology, Bern University Hospital, University of Bern, Bern, Switzerland; 4 sitem Center for Translational Medicine and Biomedical Entrepreneurship, University of Bern, Bern, Switzerland; 5 Department of Women’s and Children’s Health, Karolinska Institutet, Stockholm, Sweden

## Abstract

**Background:**

Apnea of prematurity cannot be reliably measured with current monitoring techniques. Instead, indirect parameters such as oxygen desaturation or bradycardia are captured. We propose a Kalman filter-based detection of respiration activity and hence apnea using multichannel esophageal signals in neonatal intensive care unit patients.

**Methods:**

We performed a single-center observational study with moderately preterm infants. Commercially available nasogastric feeding tubes containing multiple electrodes were used to capture signals with customized software. Multichannel esophageal raw signals were manually annotated, processed using extended Kalman filter, and compared with standard monitoring data including chest impedance to measure respiration activity.

**Results:**

Out of a total of 405.4 h captured signals in 13 infants, 100 episodes of drop in oxygen saturation or heart rate were examined. Median (interquartile range) difference in respiratory rate was 0.04 (−2.45 to 1.48)/min between esophageal measurements annotated manually and with Kalman filter and −3.51 (−7.05 to −1.33)/min when compared to standard monitoring, suggesting an underestimation of respiratory rate when using the latter.

**Conclusions:**

Kalman filter-based estimation of respiratory activity using multichannel esophageal signals is safe and feasible and results in respiratory rate closer to visual annotation than that derived from chest impedance of standard monitoring.

## Impact

- Using extended Kalman filtering applied to multichannel esophageal signals enables estimation of respiratory rate in preterm infants under clinically meaningful circumstances.
- Conventional chest impedance measurements are known to be prone to motion artifacts, which makes apnea detection unreliable. Therefore, indirect measures as oxygen desaturation are commonly taken as indicator for such events. In contrast, esophageal signals benefit from a stable electromechanical interface, making them less prone to artifacts. Integration of esophageal electrodes into a clinically required feeding tube is an additional benefit of the applied technique.
- Less artifacts in respiratory rate measurement would improve apnea detection and hence patient care.

## Introduction

Apnea of prematurity is a well-described condition in preterm infants.^1–3^ Various apnea definitions exist, commonly referring to a cessation of breathing of at least 15–20 s, potentially being associated with hypoxia and/or bradycardia.^4,5^ Subgroups such as central, obstructive, or mixed apnea are further distinguished.^5,6^ Importantly, prolonged apnea episodes leading to considerable oxygen desaturation (<80%) increase the risk of death or long-term neurodevelopmental impairment in preterm infants.^7,8^

An unresolved problem in daily clinical care on a neonatal intensive care unit (NICU) is the unreliable detection of respiratory rate (RR) with standard monitoring systems using chest impedance (CI).^9^ CI is known to be prone to motion artifacts, which may lead to false positive or false negative breath detection.^10^ Consequently, in case of apnea, an audible alarm is often not triggered by the CI monitor, but rather by accompanying monitoring systems observing oxygen saturation (SpO_2_) or heart rate (HR), i.e., when associated hypoxia or bradycardia occur.

To overcome the limitations of CI, surface diaphragmatic electromyography (EMG) has been proposed to detect RR in neonates.^11^ Surface EMG might be superior in differentiating the apnea subtypes; however, similar susceptibility of surface EMG to motion artifacts as CI have been shown.^12^ Additionally, EMG as well as CI rely on surface skin electrodes, which may harm the fragile skin of preterm infants with a subsequently increased risk of infection.^13,14^

Neurally adjusted ventilatory assist (NAVA) was developed to measure diaphragmatic activity and synchronize mechanical ventilation with the patients’ own breathing effort.^15,16^ NAVA uses commercially available gastric feeding tubes equipped with multiple electrodes, i.e., the *Edi* tube (Maquet, Getinge Group, Solna, Sweden) to detect diaphragmatic activity of the patient to trigger the ventilation. If esophageally detected respiration can be used safely and effectively for synchronization of non-invasive respiratory support in preterm infants is not yet determined due to limited data,^17^ although its feasibility has been demonstrated before.^18^

In a previous publication, we demonstrated reliable HR detection in non-ventilated preterm infants using multichannel esophageal signals from such a feeding tube.^19^ Based on the same preterm dataset, the present study aims to estimate RR from multichannel esophageal signals in non-ventilated infants. Respiratory activity is dominated by diaphragmatic motion and causes esophageal electrodes to move, as well, which manifests as baseline wander in esophageal signals. Similarly, baseline wander may result from cardiac motion and esophageal peristalsis.^19^ Due to overlapping frequency spectra of these three components of baseline wander,^20^ and further complications from catheter motion artifacts, e.g., due to coughing, simple linear (frequency-based) filters are not applicable to reliably isolate respiratory motion.^21^ For this purpose, model-based filtering, such as Kalman filters, might provide a solution. Kalman filters have successfully been applied, e.g., to denoise multichannel electrocardiogram (ECG) signals with strong interferences or RR estimation from combined surface ECG and photoplethysmography measurements.^22–24^

### Hypothesis

We hypothesize that (i) detection of RR by model-based processing of multichannel esophageal signals is safe and feasible in non-ventilated infants during daily clinical practice in the NICU and (ii) derived RR is comparable to CI respiratory monitoring during clinically meaningful events including oxygen desaturation obtained from pulse oximetry and bradycardia detected by ECG.

## Methods

### Study design and subjects

This is a prospective single-center observational study, conducted at the University Children’s Hospital Basel, Switzerland. Between July 2015 and April 2016, we prospectively included infants with a postmenstrual age between 32 and 42 weeks, after their first week of life. Inclusion criteria were a signed informed consent of the parents, need of a gastroesophageal feeding tube, and presumed stay in the NICU for >5 days. Exclusion criteria were major congenital malformations, need of vasoactive drugs, and endotracheal ventilation. Demographic and clinical data from the perinatal period to first discharge home or transfer to another hospital was collected. Documentation of possible adverse events such as tube misplacement or secondary dislocation as well as observation of skin irritation at place of external fixation was noted for all participants. All patient data were stored in a coded format.

The study was approved by the Ethical Committee of Northwestern and Central Switzerland (EKNZ: 2015-136) and was performed in accordance with the principles of the Declaration of Helsinki. The study was registered at ClinicalTrials.gov (identifier NCT02501512).

### Measurement set-up and protocol

Before onset of the study, the normal feeding tube was replaced by an *Edi* catheter conventionally used in conjunction with NAVA ventilation. This polyurethane-based tube is equipped with ten stainless steel electrodes placed at the distal end with an inter-electrode distance of 6 mm. The most proximal esophageal electrode (electrode #1) was used as ground. The second most proximal electrode (#2) served as reference. We then formed bipolar leads, referred hereafter as eECGm−2, with m depicting the more distal electrodes (#3 to #10), providing *n* = 8 esophageal leads. Esophageal signals, hereafter called *NEO* (Neonatal Esophageal Observation) signals, were captured with customized software built for this study based on MATLAB (The MathWorks^®^, Inc., Natick). Correct placement of the electrode was ensured using a graphical user interface of the software depicting esophageal ECG signal with visible amplitudes and correct feeding capability. The esophageal signals were acquired using a dedicated recorder (g.USBamp, g.tec medical engineering GmbH, Schiedlberg, Austria). The recorder amplified the unfiltered signals using direct current (DC) coupling, an input range of ±250 mV, and a sampling rate of 4800 Hz.

In addition to the esophageal electrodes, three pre-gelled surface electrodes designed to be used in preterm infants (Multi Biosensors Inc., TX) were attached to the infant’s thorax. The additional bipolar ECG, necessary for later synchronization of the *NEO* signals with the standard monitoring ECG, was amplified and simultaneously digitized in the same way as the esophageal leads and stored in the customized software.

**Fig. 1:**
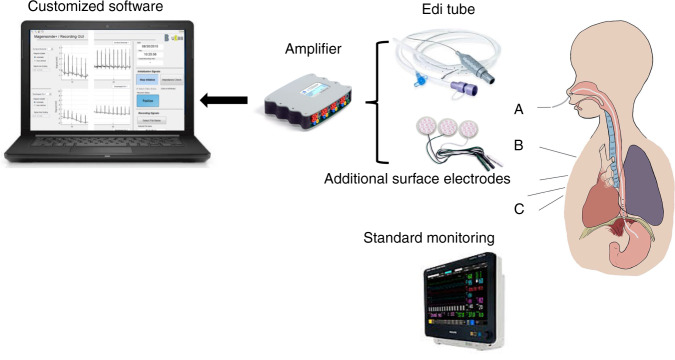
Overview of study setting. (A) Multichannel Neonatal Esophageal Observation (NEO) signal acquisition using the Edi tube, (B) simultaneous recording of surface electrocardiography. (C) *NICU* standard monitoring, including electrocardiography to measure heart rate, chest impedance measurement for the detection of respiratory rate, and pulse oximetry to measure peripheral oxygen saturation.

Standard monitoring data (hereafter called *NICU*) were stored and synchronized to *NEO* signals to ensure comparison with state-of-the-art technology. The data extraction from bedside NICU monitor screens (Philips IntelliVue MX, 700, Philips, Amsterdam, the Netherlands) was enabled using the commercially available software (ixTrend 2.0 Express, ixcellence GmbH, Wildau, Germany). The *NICU* output included the following signals and the corresponding numeric series: surface ECG and HR, CI and RR, and SpO_2_ level. An overview of the study setting can be seen in Fig. 1.

Briefly, we performed *NEO* and *NICU* measurements on 5 consecutive days in each study participant. On the first and last day, measurement duration was 24 h; on days 2–4, periods of 3 h were recorded. A detailed description of the study set-up, measurement protocol, and *NEO* signal processing has been published elsewhere.^19^

### Signal pre-processing

To be able to compare *NEO* and *NICU* signals, we first performed resampling of *NICU* data to the sampling frequency of *NEO* data and synchronized them based on QRS-peak detection from both sources to eliminate possible drift of signals. A notch-filter (50 Hz, bandwidth 1.4 Hz) was applied to the raw *NEO* data to suppress power-line interference.

**Fig. 2:**
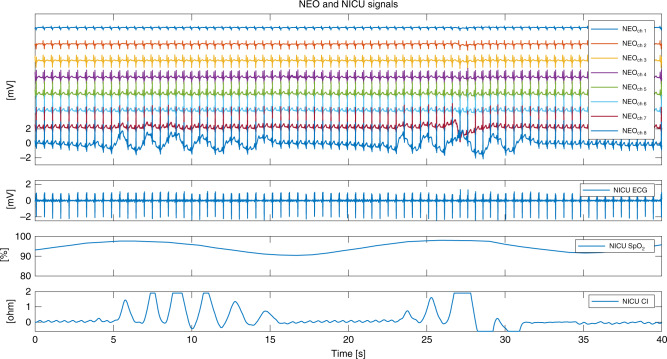
Example of *NEO* and *NICU* signals during a 40-s period. First panel: raw signal of eight esophageal leads (*NEO* channel 1: most proximal, channel 8: most distal lead), second panel: surface *NICU* electrocardiography (ECG) signal, third panel: *NICU* oxygen saturation (SpO_2_) signal, fourth panel: *NICU* chest impedance (CI) signal.

An example of synchronized data from *NEO* and *NICU* signals can be seen in Fig. 2 for the whole set-up, as well as in Figs. 1 and 2 of the Supplementary Material for good and bad signal quality, respectively.

### Data processing and labeling

Measurement periods with clinically relevant events, namely, bradycardia (values <80 beats per minute over at least 5 s) or drop in SpO_2_ (values <88% over a period of at least 5 s) based on the *NICU* numerics data output were automatically detected. We chose a short duration of hypoxia and bradycardia, as we were studying a rather healthy population of moderately preterm infants, and because many apneas are known to be of shorter duration than 20 s.^5^ For each event, a window of at least 2 min (1 min before onset of bradycardia and/or oxygen desaturation until 1 min after resolution of the event) was analyzed.

**Fig. 3:**
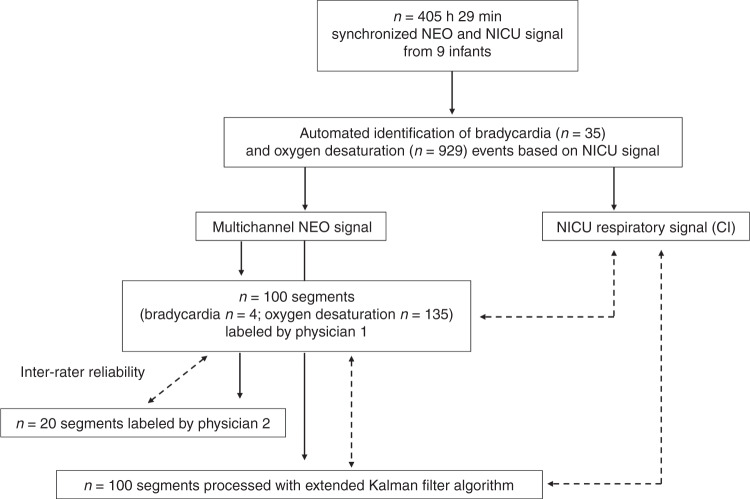
Labeling of 100 randomly chosen clinically relevant events by physicians was done by visual inspection of multichannel *NEO* signals. Solid lines: flow of data through different assessment steps; dotted lines: comparison of respiratory rate by different sources, using Wilcoxon signed-rank test and Spearman’s rank correlation coefficient for inter-rater reliability. CI chest impedance, NEO Neonatal Esophageal Observation, NICU Neonatal Intensive Care Unit.

Out of the 964 detected bradycardia and/or drop in desaturation events, 100 were selected without prior knowledge of patient characteristics or signal quality. A trained physician (C.B.) visually labeled esophageal respiratory signal in a customized software, not knowing when exactly in the segment the bradycardia/oxygen desaturation occurred and neither which condition was present. The labeling process and comparison of outcomes is illustrated in Fig. 3 and was performed as follows: out of the eight esophageal channels, the labeling physician chose the channel with the strongest respiration signal after visual inspection of an overview of all channels. The labeling of the respiratory waves was subsequently done on a zoomed window of 20 s of the selected channel. In areas with a doubtful signal, all other channels could have been considered.

Two auxiliary lines representing the 25th and 75th percentile of the median signal amplitude for each signal trace as well as the surface ECG were additionally visible on request to distinguish respiratory activity from cardiac motion, esophageal peristalsis, and catheter motion artifacts. The synchronized *NICU* respiration signal was displayed as well (see also Figs. 1 and 2 of the Supplementary Material).

All labeled segments were divided by the physician’s impression of the esophageal respiratory signal quality into one of the following groups: good, medium, and poor signal quality.

### Extended Kalman filter

In parallel to the visual annotation, the Kalman filter was applied to the same segments of clinically relevant events. The idea behind a Kalman filter is to estimate hidden time-varying process parameters based on time-correlated observational measurements. In the case of respiratory activity, neither the electrical activation nor the motion of the diaphragm is directly measurable with esophageal signals. The Kalman filter, however, estimates RR based on the surrogate marker of low-frequency electrode motion manifested as baseline wander. The baseline wander, particularly pronounced in the distal channels placed close to the diaphragm, correlates well with the respiration activity as shown in Fig. 2. To extract the RR from the multichannel esophageal signals, the respiration was modeled as a sinusoidal signal with a common time-varying frequency but independent amplitudes for each channel. Using the extended Kalman algorithm, instantaneous values of the frequency and subsequently the phase angle (frequency integrated with time) were estimated for each segment. Respiration rates RR+ and RR− were then calculated from the time differences between 0° and 180° phase crossings, respectively. The *NEO* RR was finally estimated by averaging RR+ and RR− and applying a moving mean filter with a window length of 5 s to attenuate model inaccuracies. No prior information about the location of the esophageal channels most sensitive to respiration activity was needed, as information from all channels were considered simultaneously. Details of the developed model and the extended Kalman algorithm will be published separately.

### Inter-rater reliability

To assess inter-rater reliability, we selected 5, 8, and 7 segments of poor, medium, and good signal quality, respectively, to be labeled by a second physician (K.J.) who was blinded to the results of the first labeling process.

### Statistical analysis

Statistical analysis was performed with MATLAB. For each segment of 2 min duration, we took the manual labeling of *NEO* respiratory signal by the first physician as reference. For each segment, median and interquartile range (IQR) of the difference from the reference RR (in units breaths per minute (bpm)) for the *NEO* derived by Kalman filter and *NICU* derived by CI were obtained and were defined as *deviation* and *variability*, respectively. This result can be interpreted for both *deviation* and *variability* as 0 to be the perfectly matching signal with less agreement the further away from 0. We compared the distribution of *deviation* and *variability* of the difference of RR from the reference for both methods over 100 segments. For *variability*, a cut-off of 15 bpm was defined to be clinically meaningful, since it is lower than the normal range of RR in neonates (40–60 bpm) but still of clinical relevance. To illustrate the level of agreement between the different measurement techniques, we displayed the results in a Bland Altman plot. Each observation point in the plot shows the mean RR of a time window of 5 s. The short window length was chosen to account for periodic breathing episodes which would get averaged out with larger windows.

Statistical significance was tested using a Wilcoxon signed-rank test, and adjustment for multiple comparison was done using a Bonferroni–Holm correction. We assumed a significance level alpha of <0.05 after the correction.

Inter-rater reliability was assessed using Spearman’s rank correlation coefficient, which can take a value from −1 to +1; −1 meaning opposite agreement (increasing vs. decreasing RR), 0 meaning no association between the two raters, and +1 describing perfect agreement. Values significantly higher than 0.5 are considered relevantly correlated and of ≥0.7 are considered as highly correlated. We further assessed the dependency of inter-rater reliability on visually labeled *NEO* signal quality by grouping comparison based on good, medium, and poor signal quality. Demographic factors of the subgroup of infants contributing to the selected signal episodes were compared with the overall study population using Mann–Whitney *U* test for continuous variables and Fisher’s exact test for comparison of categorical variables.

## Results

### Study population and measurements

Table 1 of the Supplementary Material shows an overview of demographics of study participants. In total, 60 measurements performed in 13 infants (*n* = 7 male) with a mean^14^ gestational age of 33.0 (28.9–35.1) weeks and birth weight of 1621 (970–2340) g were considered. We finally included a total of 31 measurements of 405.4 h duration performed in 9 infants for further signal analysis after excluding 29 measurements for reasons including transfer of the infant to another hospital (*n* = 5), transfer to another ward with different standard monitoring system (*n* = 16), inadequate signal quality or interrupted sampling (*n* = 2 for *NEO*; *n* = 6 for *NICU*). No adverse events were noted throughout the study.

Within these 31 measurements, 964 clinically relevant episodes of bradycardia (<80 beats per minute over >5 s) or oxygen desaturation (SpO_2_ < 88% over >5 s) were detected. Mean (standard deviation) duration of bradycardia and oxygen desaturation episodes was 16.6 (4.7) and 27.3 (45.9) s, respectively, with lowest values of 62.3 (2.5) beats per minute for HR and 81.3 (6.1)% for SpO_2_. In the randomly selected *n* = 100 segments of *NEO* signal, 8 of the 9 infants with complete datasets were represented, 1 infant coincidently fell out. These segments consisted of 23 episodes with good, 54 with medium, and 23 with poor signal quality, as characterized by visual inspection. Characteristics of overall study population and infants contributing to the finally selected episodes did not show any significant differences (see also Table 1 in the Supplementary Material).

### Main findings

**Fig. 4:**
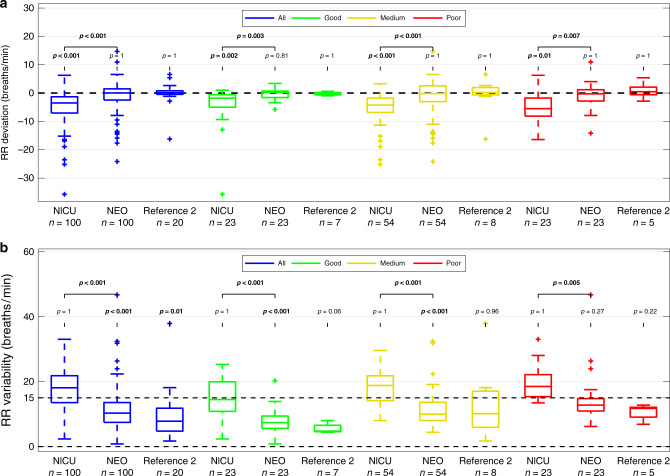
Comparison of respiratory rate from different sources. **a**, **b** Difference of *deviation* (median) and *variability* (interquartile range) of respiratory rate from the reference for different signal source annotation techniques within the segments. Manual labeling of respiratory rate on *NEO* signal taken as reference, compared to *NICU* signal using chest impedance, *NEO* signal analyzed with Kalman filter (NEO), and from Reference 2 (second manual annotation on *NEO* signal). Displayed as all example episodes (blue, *n* = 100), subgroup of visually annotated as good esophageal signal quality (green, *n* = 23), medium signal quality (yellow, *n* = 54), and of poor signal quality (red, *n* = 23). Comparison performed using Wilcoxon signed-rank test. **a** Median *deviation* of respiratory rate, **b** interquartile range *variability* of respiratory rate. A significantly better agreement between the annotation techniques and the reference, corresponding to significantly lower *variability* than the set cut-off of 15 breaths per minute (dashed line 15) was considered as clinically relevant. NEO Neonatal Esophageal Observation, NICU standard monitoring from neonatal intensive care unit, RR respiratory rate.

RR was successfully extracted by the Kalman filter in the selected segments. When all 100 segments were considered, median (IQR) RR *deviation* from manually labeled *NEO* reference was 0.04 (−2.45 to 1.48, *p* = 1.0) bpm for Kalman filter and −3.51 (−7.05 to −1.33, *p* < 0.001) bpm for *NICU*, respectively (see Fig. 4a). RR *variability* within the segments when compared to visually labeled reference was significantly lower in *NEO* Kalman-derived RR with median (IQR) 10.29 (7.46 to 13.56) bpm vs. *NICU* derived RR with median (IQR) 18.09 (13.53 to 21.83) bpm, *p* < 0.001(see Fig. 4b).

**Fig. 5:**
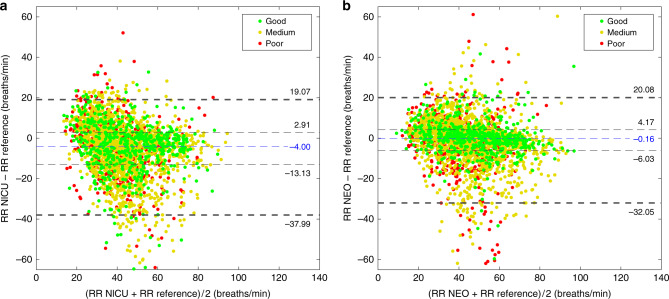
Bland Altman plot comparing *NEO* and *NICU*. **a**, **b** Bland Altman plot showing comparison between detecting respiratory rate using Reference (manual annotation of esophageal signal) and *NICU* (chest impedance from standard monitoring) (**a**) and *NEO* (extended Kalman filter based on esophageal signal) (**b**). Different esophageal signal qualities are colored as green (good), yellow (medium), and red (poor). Blue dashed line indicates median, inner black dashed lines indicate the interquartile range, and outer black dashed lines the 95% limits of agreement.

The narrower concentration of the points around the *x*-axis in the Bland Altman plot (Fig. 5) represents the smaller deviation and variability of RR *NEO* from visual annotation compared to *NEO* analyzed by Kalman filter (Fig. 5b) than when compared to *NICU* (Fig. 5a).

### Subgroup analysis based on signal quality

Subgroup analysis by visually categorized esophageal signal quality did not show significant differences in *NEO* RR using visual labeling vs. Kalman filtering approach (*p* = 0.81 for good and 1.0 for medium and poor signal quality). In contrast, significant *deviations* between visual labeling and *NICU* RR could be observed irrespective of esophageal signal quality (*p* = 0.002 for good, *p* < 0.001 for medium, and *p* = 0.01 for bad signal quality). RR *variability* showed significantly better agreement of the *NEO* Kalman filtering approach with manual labeling within segments of good and medium quality (*p* < 0.001, corresponding to a difference lower than the set cut-off of 15 bpm within the segments) and a non-significant difference to 15 bpm variability in segments with poor signal quality (*p* > 0.05). When comparing *variability* of visually labeled *NEO* RR and RR derived by *NICU*, all segments showed differences exceeding the pre-defined cut-off of 15 bpm, irrespective of signal quality (*p* = 1.0, see also Fig. 4a, b).

### Secondary findings

Inter-rater reliability on visual *NEO* respiration labeling assessed using Spearman’s rank correlation coefficient showed a high correlation with median (IQR) 0.8 (0.51–0.87) bpm when looking at all 20 selected segments. Figure 3 of the Supplementary Material also depicts the decreasing agreement between the two raters in case of lower signal quality.

## Discussion

Results from this study indicate that detection of esophageal-derived respiration is safe and feasible in non-ventilated preterm infants. The median deviation of RR estimation was 0.04 bpm in periods of clinically meaningful events such as bradycardia and oxygen desaturation and was not considerably altered during periods of low esophageal signal quality.

### Interpretation of results

Automatically extracted RR from *NEO* signal using an extended Kalman filter is significantly closer to the visually labeled RR in terms of *deviation* (median values) as well as *variability* (IQR), than RR derived by *NICU*. The better agreement was particularly present in segments of good signal quality. These results are confirmed in the Bland Altman plot, with a higher concentration of points near the *x*-axis when comparing *NEO* than when comparing *NICU* to the visually labeled RR. Our results indicate a higher robustness of the esophageal method to signal perturbations. Most likely, esophageal electrodes benefit from a more stable electrochemical environment within the esophagus and the proximity to the diaphragm, making them less prone to motion artifacts. Such artifacts resulting from catheter motion, e.g., due to esophageal peristalsis or head movements have not provoked signal saturation due to the DC-coupling amplifier^19^ and thus can subsequently be attenuated by the Kalman filter. In contrast, CI-derived signals from surface electrodes suffer from artifacts due to skeletal muscle activity or electrode/cable motion. Such artifacts often manifest as saturation of the impedance signal in case of high skeletal muscle activity (see also Fig. 2 of the Supplementary Material), making a reliable detection of RR during those periods cumbersome. This could lead to under-recognition of apnea when relying on CI as a signal source.

The association of lower inter-rater reliability and bad signal quality is understandable, given the more difficult task of manual labeling in case of noisy and artifact-rich signals. The fact that esophageal electrodes were only attached over a total length of 6 cm at the distal end of the *Edi* tube could lead to already significant signal degradation in case of minor dislocation of the catheter. The extended Kalman filter approach compensated this with its ability to use signal information from all the esophageal channels to extract respiration activity. This estimation is based on “predictions” calculated from the assumed system dynamics (sinusoidal behavior of respiratory activity) and the *optimal* “corrections” to the estimated states based on the observed values. The weights (the process and measurements noises in the Kalman filter), which determine the extent of these “corrections,” are calculated at each time step from the averaged quantities of the past estimations. Indeed, the predictive model helps to suppress abrupt changes in the estimated parameters due to non-stationary signal disturbances, whereas the continuous adjustment of the weights helps in adapting to changes in signal characteristics due to catheter movements.

### Comparison with previous literature

The limited accuracy of today’s standard respiratory monitoring on a NICU using CI is well known.^11^ Apnea is therefore only indirectly measured, i.e., assumed to be the cause of subsequent events, such as bradycardia or oxygen desaturation. Several research groups recently tried to improve monitoring techniques to better depict the respiratory pattern of newborns. Kraaijenga et al. compared surface diaphragmatic EMG measures with CI in infants and found comparable results, with similar susceptibility to surface electrode motions.^11^ Other studies focused on the usability of esophageal signals applying the conventional NAVA interface in both ventilated^15,16^ and non-ventilated preterm infants.^18^ However, a recently published systematic review concludes that the evidence about usefulness of NAVA in infants on non-invasive respiratory support is too limited to draw firm conclusions about its clinical utility yet.^17^

In contrast to the described EMG-based techniques, the applied Kalman filter extracts respiratory activity from diaphragm movement, which manifests as low-frequency baseline wander of the esophageal signal with pronounced magnitudes. Commercially available NAVA respiration detection is based on the EMG of the diaphragm (EMGdi), which usually suffers from low magnitude in the esophageal leads, similar to surface EMG measurements. Reliable detection of esophageal EMGdi is further dependent on the relative position between electrodes and diaphragm, which is difficult to maintain over long observation periods. With our method, we expect the motion signal to be less spatially localized and therefore less dependent on the electrode–diaphragm distance. Besides, the motion signal is easily distinguishable from higher frequency components such as the ECG, EMG from other muscles, and the power-line interference, all turning a reliable detection of EMGdi difficult.

Other research groups are focusing on non-contact monitoring techniques, which would reduce the risk of skin irritation when compared to standard monitoring or surface EMG. Most of these studies, however, are performed in healthy adults or under well-controlled research environments during a limited time period, which makes the findings less applicable for continuous monitoring in a NICU. One study performed over several consecutive days in a NICU using high-resolution video-based detection of different vital signs could demonstrate high agreement with standard monitoring for detection of HR.^25^ However, for RR it seemed to be less accurate based on the visual data reported in this study. Unfortunately, the authors did not provide a statistical analysis of the RR data, therefore a statement on accuracy of video-monitoring cannot be made. Besides, during periods of spontaneous movements of infants or lack of visible skin surface, due to, e.g., clothing, instrumentation, shielding of patients, or as a results of humidity in an incubator, vital signs cannot be measured accurately.^25^ Another group used piezoelectric sensors between the infants and the mattress to measure an acoustic signal of the cardiorespiratory activity. Again, HR was detected with high robustness and especially well in quiet episodes. RR on the other hand was comparable to standard monitoring during quiet episodes but corrupted during motion or when mechanical ventilator noise disturbed the signal.^26^ The mentioned techniques are promising especially in a controlled research environment but might be difficult to apply in daily clinical practice in a NICU. Especially in critical situations, accurate monitoring is key, but care-givers or instruments might block the view to the body of the patients or introduce noise that disturbs acoustic signals. Although the *NEO* approach estimates RR from diaphragm motion, the above limitation does not hold as the signal is acquired from sensors incorporated into the body. The multichannel esophageal signals are acquired with electrodes mounted on a gastric feeding tube, a commonly required long-term requisite in preterm infants. Thus, by combining a feeding tube with sensors to detect vital signs, skin irritations and lesions may be mitigated. Some studies used esophageal manometry to estimate respiration activity; however, these did not report any comparison with standard monitoring.^27–29^ Additionally, currently available manometry catheters are too big and inflexible to be used for long-term monitoring and feeding as needed in a NICU.

The usage of tiny skin-interface sensors for wireless measurements of various vital signs was proposed lately in a report of two pilot studies.^30^ The obtained vital signs were not only comparable to standard monitoring techniques but also provided additional information on hemodynamic and cardiovascular health as well as on activity and position of the study participants.^30^ Although the respiration signal was estimated based on surface ECG and skin vibration measurements using electrodes without cables, motion artifacts could not be completely suppressed.

### Clinical impact

Correct detection of critical situations resulting from a prolonged cessation of breathing is of major importance in a NICU. The monitor should detect all clinically relevant episodes and raise as little false positive alarms as possible such that alarm fatigue in health care staff can be prevented.^31,32^ One possible way to reduce false alarms would be to incorporate additional sensor modalities, which, however, until now are still a matter of ongoing research as shown by Muroi et al.^33^ Another attempt would be to improve the primary detection of the signal of interest by reducing noise and artifacts at the sensor side, as we aimed to test with the acquisition of multichannel esophageal signals. The proposed *NEO* method captures changes of RR instead of indirect measures as bradycardia or desaturation. This direct measure would lead to earlier recognition of potentially dangerous situations. A more accurate respiration signal would also lead to a better understanding of apneas, which could finally improve patient care and consequently reduce health care costs.^34,35^

Esophageal electrodes require the installation of a gastric tube, which bear a minimal risk of local irritation or even infection. If improperly placed, the tube can also affect breathing and HR. Nevertheless, patients in a NICU usually require a feeding tube due to immature swallow–breath coordination anyway and esophageal electrodes do not add any additional risk. The replacement of the surface sensors by esophageal electrodes would additionally decrease the number of wires attached to the patient’s body, leading to easier patient care as well as bonding while simultaneously protecting the infant’s skin.

However, to date a real-time implementation of RR estimation based on multichannel esophageal signals has not yet been possible but would first have to be tested in a prospective clinical study.

### Implication for future research

Mathematical characteristics of vital signs have been increasingly studied among preterm infants, mainly for its predictive value as marker for early detection of sepsis.^36–38^ The main focus so far has been on HR characteristics, as the activity of the heart is easier to detect with common standard surface monitoring and results are therefore more reliable. Some studies have additionally assessed respiratory patterns, but mainly within short, controlled measurement episodes or selected patient populations, limiting the generalizability of findings in daily clinical practice.^39,40^ Therefore, as reliable data about maturation of breathing (ir)regularity is missing, a lot of physiological knowledge is still to be elaborated. There might be plenty of prognostic physiological information within the breathing pattern of neonates, potentially useful for prediction of both acute and more prolonged outcomes,^39,41^ which is not accessible from today’s standard monitoring. This highlights the necessity of an accurate technique to continuously estimate RR in daily clinical practice. We therefore believe that the present study demonstrates a valuable basis for further investigating this research route.

A limitation of CI is its inability to detect obstructive apnea. In the current analysis, we did not test to detect obstructive apnea. However, it is conceivable that the changes in diaphragm activity during obstructive apnea manifest as a change of characteristics of motion (e.g., instantaneous amplitude or frequency), which are estimated by the Kalman filter with a high degree of resolution.

Whether the *NEO* technique can be adopted to even more premature infants or to infants on mechanical ventilation, and whether real-time application would lead to similar results, needs to be tested in future studies. Additionally, improvements of the used catheter, e.g., by enlarging the electrode surface and number of electrodes to cover a wider section of the esophagus, could improve esophageal signal quality and hence the reliability of the method. Also, incorporating more sensing modalities (e.g., pressure and acceleration sensors) into a single gastric catheter can increase the reliability of the estimated parameters. The Kalman filter approach can be extended to include signals from multiple sensors and thus might benefit from the differences in their sensitivities for various physiological processes. Technological advances in the miniaturization of sensors might enable their integration into the *NEO* catheter extending the diagnostic capability similar to soft skin interfaces.^30^

### Strengths and limitations

A major difficulty when assessing the *NEO* technique was the lack of a gold-standard to measure RR accurately during daily clinical care in non-ventilated infants given the fact that CI is known to be prone to motion artifacts and signal saturation. There are several ways to assess more detailed ventilation parameters in non-ventilated infants during study settings using lung function measurements.^42^ But until now these techniques are only applied in controlled research environments and special study settings. We therefore visually inspected and manually labeled *NEO* signals to assure correct detection by the applied Kalman filter, additionally to the comparison of *NEO* vs. *NICU* signals. It must be considered that during manual labeling of *NEO* signals the *NICU* respiration signal was visible. Besides, we did perform manual labeling of the esophageal signal and later compared both *NEO* Kalman and *NICU* data with this visually labeled method. Both approaches could bias the results.

As the electrodes are placed in the esophagus, interference (e.g., due to peristalsis) with the breathing signal and therefore false high or false low RR is possible. As esophageal peristalsis can be seen as a motion moving from proximal to distal, it affects all electrodes consecutively with a small time shift and usually shows a larger amplitude than respiration activity. We therefore think that this did not affect the visual labeling of RR and hence comparison between *NEO* and *NICU* substantially.

A further limitation is the small number of patients included in this study. Given the study design to test safety and feasibility of the *NEO* technique and the high number of measurement hours, as well as clinically relevant episodes that could be studied, we expect that a larger population would not considerably have affected the study results.

### Conclusion

Multichannel esophageal measurement of RR in non-ventilated infants is feasible and safe. Applying an extended Kalman filter to the esophageal signal results in estimation of RR closer to visually annotated RR than that of RR derived from standard CI monitoring during meaningful episodes of bradycardia and oxygen desaturation. Thus, esophageal respiration signals might be more reliable due to their proximity to the organ of interest and robustness against motion artifacts or sensor displacements compared to surface electrodes. Applicability of real-time monitoring of RR using multichannel esophageal signals and its non-inferiority compared to standard surface monitoring during daily clinical practice need to be tested in future prospective studies.

## Supplementary information

Supplementary_Material
