# Supplementary material for: Multichannel esophageal signals to monitor respiratory rate in preterm infants
Source: Pediatr Res. 2021 Oct 2;91(3):572–80. doi: 10.1038/s41390-021-01748-4 (PMC8487228; doi:10.1038/s41390-021-01748-4)
Supplement: Supplementary file 1 — Supplementary_Material [file 41390_2021_1748_MOESM1_ESM.pdf]

Supplementary Material

Multichannel Esophageal Signals to Monitor Respiratory Rate in Preterm Infants

Table 1, Demographic information of study participants

**Variable**	**Overall study population (n=13 infants)**	**Infants contributing to 100 selected episodes (n= 8 infants)**	**p-value**
GA (w)	33.0 (1.9)	32.9 (1.5)	0.379
PMA (w)	34.3 (1.3)	34.0 (0.9)	0.340
BW (g)	1621 (428)	1724 (422)	0.272
BW z-score	-1.1 (1.1)	-0.7 (0.7)	0.189
Sex, male	7 (54)	6 (75)	0.103

Continuous variables are displayed as mean (standard deviation) and compared using Mann-Whitney U test; sex is displayed as numbers (%) and compared using Fisher's exact test. **Abbreviations:** BW: birth weight, g: gram, GA: gestational age, PMA: postmenstrual age (gestational age plus postnatal age), w: weeks.

Figure 1 supplementary material

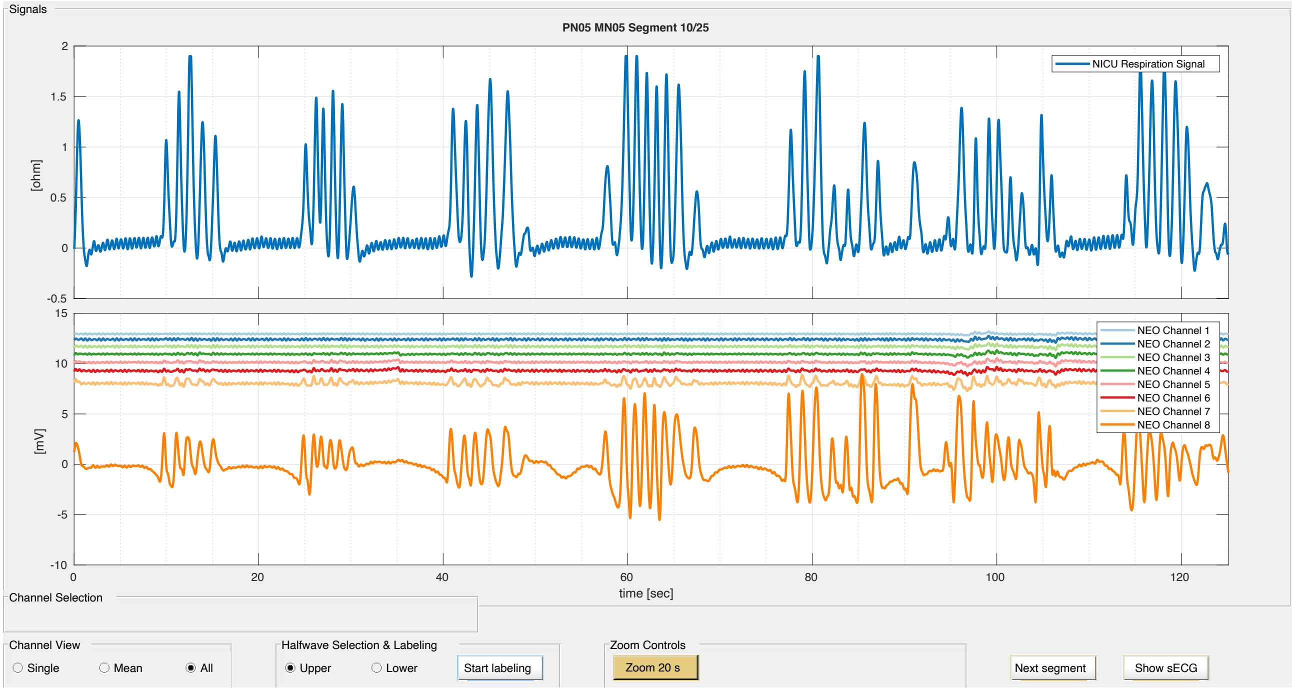

Graphical interface of the labeling software depicting an example of good signal quality, upper panel showing *NICU* respiratory signal, lower panel showing esophageal channels 1-8 (proximal to distal) with pronounced respiratory activity in more distal channels.

Figure 2 supplementary material

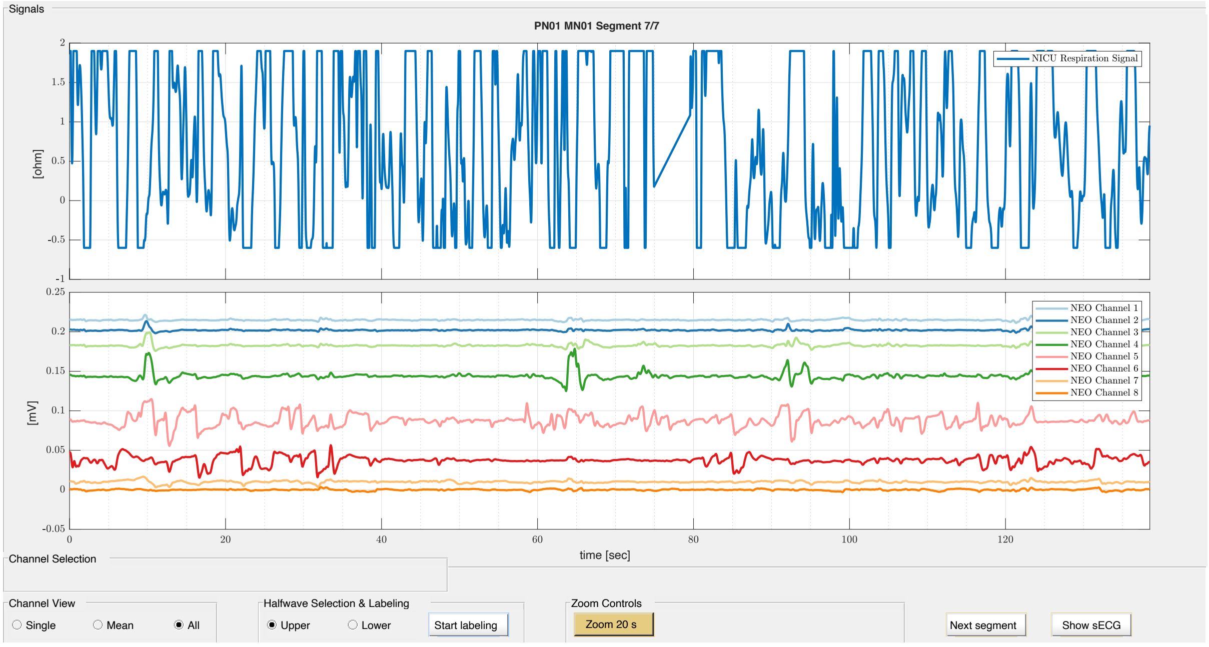

Graphical interface of the labeling software depicting an example of bad signal quality, upper panel showing *NICU* respiratory signal, lower panel showing esophageal channels 1-8 (proximal to distal).

Figure 3 supplementary material

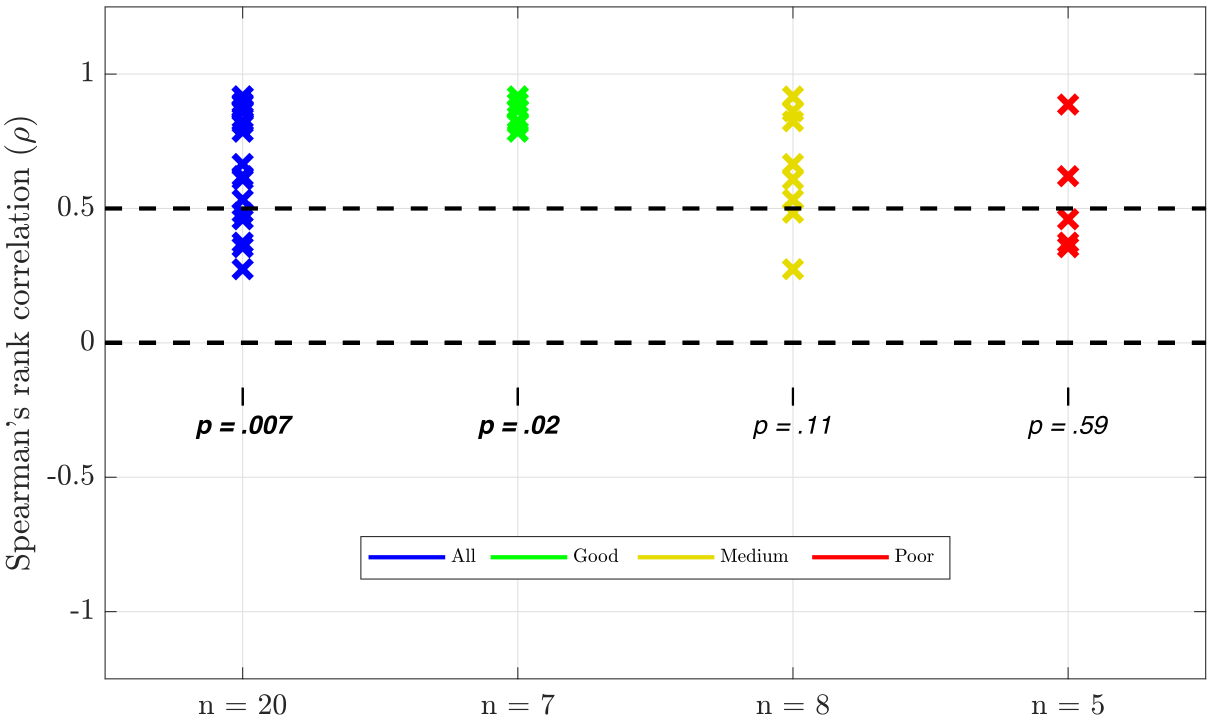

Interrater reliability of manual labeling of episodes of respiratory rate with differing signal quality on *NEO* signal using Spearman's rank correlation coefficient. Displayed are all assessed episodes (blue, n=20), subgroup of visually annotated as good esophageal signal quality (green, n=7), medium signal quality (yellow, n=8), and of poor signal quality (red, n=5). Dashed lines y-axis at value 0: indicating no interrater agreement, at 0.5: indicating cut-off for measure of significance. P-values indicate the probability of reaching significant rank correlation between raters (>0.5).
